# Postcollisional cooling history of the Eastern and Southern Alps and its linkage to Adria indentation

**DOI:** 10.1007/s00531-016-1367-3

**Published:** 2016-07-13

**Authors:** Bianca Heberer, Rebecca Lee Reverman, Maria Giuditta Fellin, Franz Neubauer, István Dunkl, Massimiliano Zattin, Diane Seward, Johann Genser, Peter Brack

**Affiliations:** 1 Department of Geography and Geology, University of Salzburg, Hellbrunner Str. 34, 5020 Salzburg, Austria; 2 Geological Institute, ETH Zürich, Sonneggstrasse 5, 8092 Zurich, Switzerland; 3 Department of Earth and Planetary Sciences, University of California, Berkeley, Berkeley, CA 94720 USA; 4 Institute for Geochemistry and Petrology, ETH Zürich, Clausiusstrasse 25, 8092 Zurich, Switzerland; 5 Geoscience Center, University of Göttingen, Goldschmidtstrasse 3, 37077 Göttingen, Germany; 6 Department of Geosciences, University of Padua, Via G. Gradenigo 6, 35131 Padua, Italy; 7 School of Geography, Environment and Earth Sciences, Victoria University of Wellington, PO Box 600, Wellington, 6012 New Zealand

**Keywords:** Southern and Eastern Alps, Low-temperature thermochronology, Adria indentation, Exhumation

## Abstract

Indentation of rigid blocks into rheologically weak orogens is generally associated with spatiotemporally variable vertical and lateral block extrusion. The European Eastern and Southern Alps are a prime example of microplate indentation, where most of the deformation was accommodated north of the crustal indenter within the Tauern Window. However, outside of this window only the broad late-stage exhumation pattern of the indented units as well as of the indenter itself is known. In this study we refine the exhumational pattern with new (U–Th–Sm)/He and fission-track thermochronology data on apatite from the Karawanken Mountains adjacent to the eastern Periadriatic fault and from the central-eastern Southern Alps. Apatite (U–Th–Sm)/He ages from the Karawanken Mountains range between 12 and 5 Ma and indicate an episode of fault-related exhumation leading to the formation of a positive flower structure and an associated peripheral foreland basin. In the Southern Alps, apatite (U–Th–Sm)/He and fission-track data combined with previous data also indicate a pulse of mainly Late Miocene exhumation, which was maximized along thrust systems, with highly differential amounts of displacement along individual structures. Our data contribute to mounting evidence for widespread Late Miocene tectonic activity, which followed a phase of major exhumation during strain localization in the Tauern Window. We attribute this exhumational phase and more distributed deformation during Adriatic indentation to a major change in boundary conditions operating on the orogen, likely due to a shift from a decoupled to a coupled system, possibly enhanced by a shift in convergence direction.

**Electronic supplementary material:**

The online version of this article (doi:10.1007/s00531-016-1367-3) contains supplementary material, which is available to authorized users.

## Introduction

**Fig. 1:**
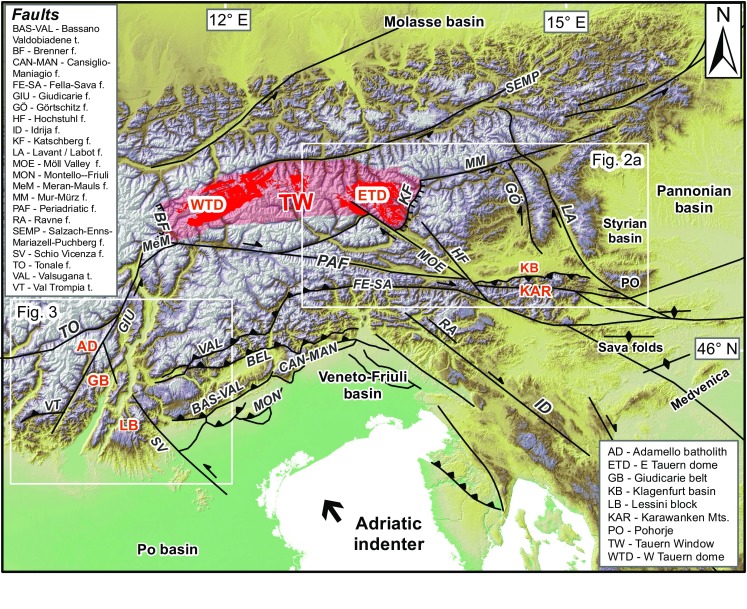
DEM of the Southern Alps, Eastern Alps and northern Dinarides displaying major faults. The *dark red* signature within the Tauern Window denotes outcropping “Zentralgneiss” of the Tauern subdomes. *Frames* denote outline of Figs. 2a and 3

Late-orogenic indentation by rigid lithospheric plates and microplates into a weaker continent leads to postcollisional shortening, lithospheric thickening, vertical and lateral extrusion and erosion (e.g., Robl and Stüwe 2005; Tapponnier et al. 1986). The European Eastern Alps are a prime example of microplate indentation (Ratschbacher et al. 1991). Their Late Neogene geodynamic framework is influenced primarily by the ca. NW-ward motion of the Adriatic plate and its counterclockwise rotation with respect to Europe, which resulted in an oblique, dextral transpressional setting (e.g., Caputo and Poli 2010; Scharf et al. 2013). The northern edge of the Adriatic indenter roughly corresponds to the Periadriatic fault system (PAF, Fig. 1), the largest, most important discontinuity and the main structural divide of the European Alps. The PAF system is offset by the sinistral NE–SW trending transpressive Giudicarie fault, which defines the western border of the eastern Adriatic indenter (sensu Handy et al. 2014), i.e., the still northward moving triangular northeastern part of the Southalpine block that indented the Eastern Alps (also labeled North Adriatic indenter by, e.g., Massironi et al. (2006), Southalpine indenter by, e.g., Pomella and Stipp (2012), or Dolomites indenter by Frisch et al. (2000), among others). The onset of indentation occurred at ca. 23–21 Ma (Pomella and Stipp 2012; Scharf et al. 2013).

**Fig. 2:**
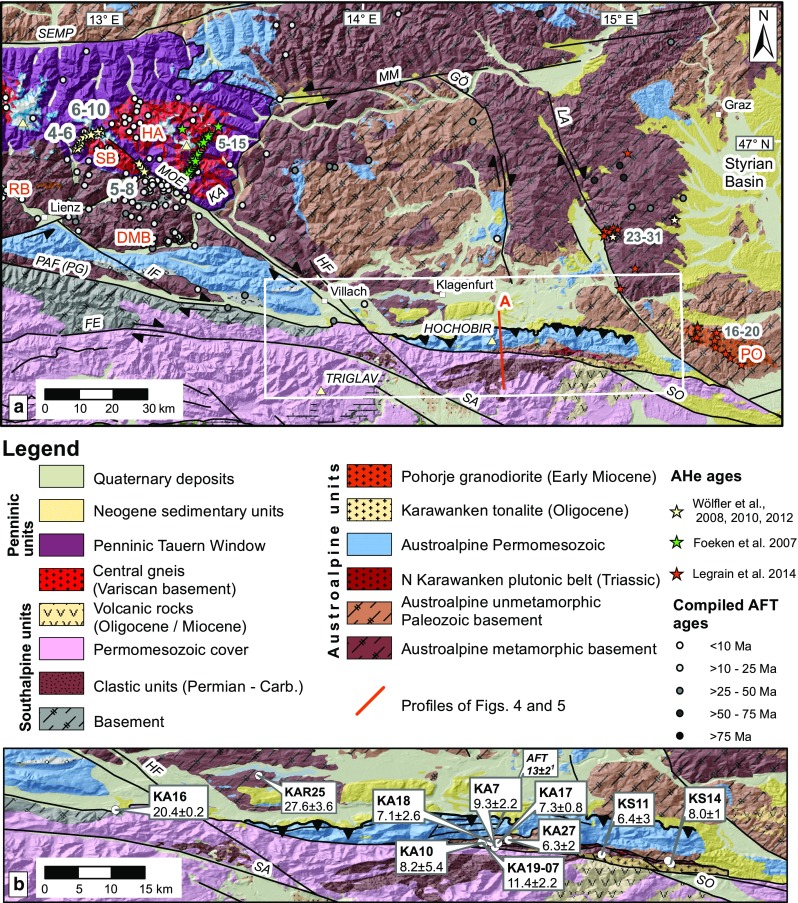
**a** Geological map of the Eastern and Southern Alps bordering the easternmost PAF and the Karawanken Mts. illustrating major faults and summarized low-temperature thermochronology data. AFT ages were compiled from Bertrand (2013), Hejl (1997), Staufenberg (1987), Wölfler et al. (2008, 2010, 2012). *DMB* Drau-Möll block, *FE* Fella fault, *GÖ* Görtschitz fault, *HA* Hochalm–Ankogel subdome, *HF* Hochstuhl fault, *IF* Isel fault, *KA* Katschberg fault, *LA* Lavant/Labot fault, *MM* Mur-Mürz fault, *MOE* Möll Valley fault, *PAF* Periadriatic fault, *PG* Pusteria–Gailtal fault segment of the PAF, *PO* Pohorje, *RB* Rieserferner block, *SA* Sava fault, *SB* Sonnblick subdome, *SEMP* Salzach–Enns–Mariazell–Puchberg fault, *SO* Sostanj fault. *Frame* denotes outline of (**b**). **b** New AHe ages with 2*σ* error for the Karawanken Mountains. Referenced AFT age from ^1^Nemes (1996)

**Fig. 3:**
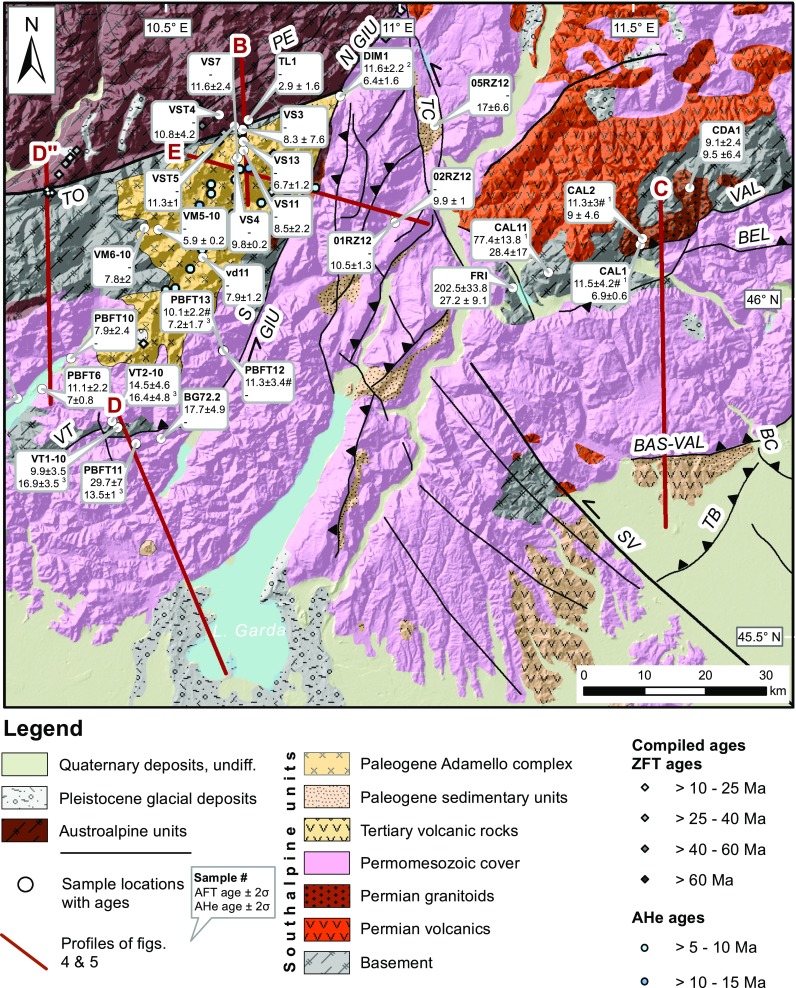
Geological map of the Southalpine units targeted for low-temperature thermochronology. New and published AFT and AHe ages with errors given as 2*σ* are shown. Labeled published ages are from ^1^Zattin et al. (2003, 2006); ^2^Pomella et al. (2011) and ^3^Reverman et al. (2012). #-Sample failed Chi-square test indicating significant dispersion in ages. ZFT ages were compiled from Pomella et al. (2011, 2012), Stipp et al. (2004), Viola (2000), Viola et al. (2001, 2003). Compiled AHe data (unlabeled) come from Reverman et al. (2012). Geology is based on the geological map of Italy, scale 1:1,250,000 (2005) and the structural model of Italy, scale 1:500.000 (Bigi et al. 1990). *BAS-VAL* Bassano–Valdobiadene thrust, *BC* Bassano–Cornuda thrust, *BEL* Belluno line, *N GIU* North Giudicarie line, *PE* Pejo fault, *S GIU* South Giudicarie line, *SV* Schio–Vicenza fault, *TB* Thiene–Bassano thrust, *TC* Trento–Cles fault, *TO* Tonale fault, *VAL* Valsugana thrust, *VT* Val Trompia thrust

The bulk of Adriatic plate motion was transferred into differential deformation north and south of the PAF system. Exhumation and deformation north of the PAF primarily occured in the axial zone of the orogen in the Tauern Window, where between 23 and 21 Ma deep, high-pressure units (the Penninic nappes) started to exhume to shallow crustal depths via orogen-parallel extension (e.g., Behrmann 1988; Scharf et al. 2013) (Figs. 1, 2). Exhumation continued until the Late Miocene leading to the formation of large-amplitude folds and domes in the Tauern Window (e.g., Schmid et al. 2013). South of the PAF system, limited exhumation from shallow crustal levels (Zanchetta et al. 2015; Zattin et al. 2006) occurred, and throughout the Oligo-Miocene most deformation was accommodated by progressive southward thrust propagation and transpressional shear along the indenter margin (Picotti et al. 1995; Pomella and Stipp 2012; Prosser 1998; Viola et al. 2001). West of the Giudicarie fault, thermochronological data from the Adamello complex, the largest Tertiary intrusion of the Alps, record rapid uplift and exhumation from a shallow depth (~2 km) between 8 and 6 Ma (Reverman et al. 2012) (Fig. 3). Much less is known about the exhumation pattern at the eastern and northeastern immediate front/rim of the eastern Adriatic indenter. For the indented units north of the PAF, in particular SE and E of the Tauern Window, age data are sparse and only the broad exhumation pattern is known (Hejl 1997; Staufenberg 1987; Wölfler et al. 2012) (Fig. 2a), although the Eastern Alps are tectonically more active than the Western and Central Alps due to ongoing indentation (e.g., Battaglia et al. 2004). Except for a single apatite fission-track (AFT) age from the Karawanken tonalite (Nemes 1996) (Fig. 2b), no low-temperature thermochronological data exist for the eastern segment of the PAF system, the first focus of this study. The published age record is more abundant for the western part of the leading edge of the eastern Adriatic indenter, but mostly limited to FT data. Along the NW corner of the indenter fission-track data on apatites and zircons (ZFT) portray a complex cooling pattern with a corridor of young Miocene ZFT ages (Pomella and Stipp 2012; Viola et al. 2001). Thermochronological data from within the indenter are again scarce due to limited availability of suitable lithologies. AFT dating of Southalpine crystalline basement in the hanging wall of a major thrust (Valsugana thrust, Fig. 3) yielded evidence for prominent Late Miocene cooling and exhumation (Zattin et al. 2006), but no constraints exist for other major structural units and new low-temperature thermochronological data are needed to bridge this gap. Thus, our second focus is on the Southern Alps, from where we present new AFT and apatite (U–Th–Sm)/He (AHe) ages that complement and enhance previous work. In particular, we focus on the Giudicarie belt and surrounding regions corresponding to the western boundary zone of the eastern Adriatic indenter.

Our goal is to capture the spatial and temporal variability of the vertical component of extrusion associated with microplate indentation and ongoing convergence during the late stages of Alpine orogeny. A better understanding of the magnitude and timing of uplift will also shed light on the discussion on the topographic evolution of the Alps (e.g., Hergarten et al. 2010; Robl et al. 2015) and its potential coupling to climate and deep-seated tectonic processes beneath the Alps (e.g., Baran et al. 2014; Cederbom et al. 2004; Fox et al. 2015; Herman et al. 2013). By recombining our new age information with the published record from the Eastern and Southern Alps and the clastic record from the peripheral foreland basin of the Karawanken Mountains we find a young, latest Mid-Miocene but predominantly Late Miocene exhumation pulse as a first-order feature of the cooling pattern. This adds a second phase of exhumation and shortening to the postcollisional evolution of the Eastern Alps, following an initial, well-known stage of Early to Middle Miocene stationary, large-amplitude exhumation concentrated in the Tauern Window (e.g., Luth and Willingshofer 2008). The spatial evolution of exhumation through time is discussed in light of Adriatic indentation.

## Geological background and tectonic setting of the study areas

After the Cretaceous-to-early-Paleogene subduction and closure of the Piemontais-Ligurian (“Penninic”) ocean, the Eocene-to-Oligocene collision of the stable European continent and the Adriatic microplate resulted in the building of the European Alps (e.g., Handy et al. 2010). The Adriatic indenter, i.e., the northern promontory of the Adriatic microplate (Southern Alps) had only experienced lower-greenschist-facies Alpine overprint (e.g., Spalla and Gosso 1999) and acted as a strong indenter (Robl and Stüwe 2005; Willingshofer and Cloetingh 2003). Intra-orogenic N–S shortening was largely compensated by orogenic thickening due to nappe stacking, e.g., by thrusting along a crustal-scale shear zone known as sub-Tauern ramp (Gebrande et al. 2002; Lammerer et al. 2008), followed by large-scale doming and exhumation (e.g., Favaro et al. 2015). In the Eastern Alps maximum amounts of collisional shortening occurred in the western part of the Tauern Window (Rosenberg et al. 2015). ZFT ages mostly range between 18 and 12 Ma in the Tauern Window (Bertrand et al. 2015 and references therein) and are older only in its SE corner (Dunkl et al. 2003; Staufenberg 1987). There, AFT and AHe ages are between 23 and 7 and 15 and 5 Ma, respectively (Foeken et al. 2007; Wölfler et al. 2012) (Fig. 2a).

The more than 700-km-long PAF is the most outstanding fault system of the Alps. It juxtaposes the N-vergent part of the orogen to the Southalpine retrowedge and separates terrains with distinct paleogeographic, magmatic and metamorphic development (e.g., Laubscher 1983; Schmid et al. 1987), which were, however, closely coupled since the Miocene (e.g., Massironi et al. 2006). The PAF delimits the SSE-vergent fold and thrust belt of the eastern Southern Alps to the north. The Giudicarie belt and the relatively undeformed Lessini foreland block represent the western, the NW–SE trending dextral Idria and Ravne faults (Fig. 1) its eastern boundary. The Southalpine fold and thrust belt east of the Adamello complex mainly developed during polyphase Neogene contraction and inversion of the Adriatic passive margin (Zampieri and Massironi 2007). The most important tectonic features of the eastern Southern Alps are from N to S the Valsugana, Belluno, Bassano and Montello thrust sheets, involving both basement and Permo-to-Cenozoic cover rocks. Frontal thrusts are linked via the NW–SE trending Schio-Vicenza and N–S trending Trento-Cles strike-slip faults to the Giudicarie belt (Massironi et al. 2006) (Figs. 1, 3).

The PAF system comprises various segments, i.e., from W to E the Canavese, Tonale, Giudicarie and Pusteria–Gailtal faults and its extension into the Karawanken Mountains (Fig. 1). This study targets two key areas in the vicinity of the PAF system at the leading northern and western edge of the eastern Adriatic indenter (Fig. 1): (1) the easternmost PAF segment within the Karawanken Mountains and (2) the central-eastern Southern Alps. In the following, we give a brief overview for those major units targeted for low-temperature thermochronological dating.

### Eastern Periadriatic fault/Karawanken Mountains

**Fig. 4:**
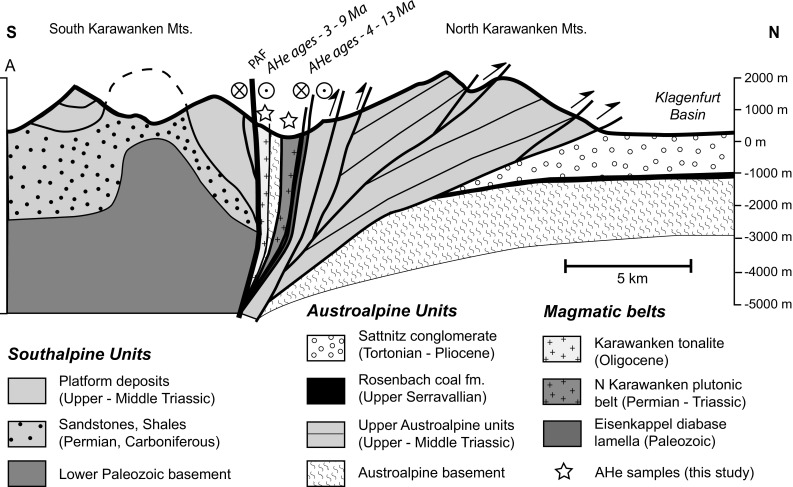
S–N section across the Karawanken flower structure (modified after Nemes et al. 1997). Trace of the section is indicated in Fig. 2

The eastern PAF system is trisected (Fig. 2): (1) A straight western segment coincides with the easternmost Pusteria–Gailtal fault. (2) The central segment is limited by the Hochstuhl–Möll Valley fault system and Lavant (Labot) fault which both offset the PAF system (ca. 4–6 km displacement, respectively, ca. 10–15 km). A positive flower structure straddles the PAF, which separates the distinct North and South Karawanken units (Laubscher 1983; Polinski and Eisbacher 1992; Tollmann 1985) (Fig. 4). (3) In the eastern segment, east of the Lavant fault, the PAF juxtaposes the Pohorje basement in the north and the Sava fold area in the south. The Sava folds were deformed by N–S shortening during Middle and Late Miocene times, leading to cooling below 110 °C from 10 to 15 Ma (Sachsenhofer et al. 2001). East of the Lavant fault, the PAF system is buried beneath the Tertiary sediments of the Pannonian basin (Fodor et al. 1998).

The brittle dextral Hochstuhl–Möll Valley fault system links the eastern PAF with the Tauern Window (Figs. 1, 2). Structural and metamorphic basement domes of Variscan granitoids (“Zentralgneis” of the Sonnblick and Hochalm subdomes, Figs. 1, 2) within the eastern Tauern window are transected by the Möll Valley fault, which also transects the eastern PAF system (Figs. 1, 2) (Kurz and Neubauer 1996; Polinski and Eisbacher 1992; Scharf et al. 2013). During the Early-to-Middle Miocene extrusion the Möll Valley fault accommodated up to 25 km of displacement (Kurz and Neubauer 1996; Scharf et al. 2013; Wölfler et al. 2008).

In the central segment, the North Karawanken unit was overthrust onto the flexural Klagenfurt basin (Figs. 2, 4). This intra-orogenic basin comprises a more than 1000-m clastic sequence ranging from fine-grained, coal-bearing early Sarmatian (Serravalian) deposits (Klaus 1956; Tollmann 1985) to coarse-grained Pontian (Late Messinian) deposits and Pliocene to possible Pleistocene conglomerates (for details see Nemes et al. 1997 and references therein). Several large and small calc-alkaline intrusions and mafic dike swarms of Oligocene age, that were emplaced due to the break-off of the European slab (von Blanckenburg and Davies 1995), are aligned along the PAF system. The segment of the Karawanken Mountains targeted for sampling displays narrow ca. E-W trending bands of Permotriassic Eisenkappel granite (North Karawanken plutonic belt), Paleozoic metasedimentary rocks and ductilely deformed Oligocene Karawanken tonalite in the immediate vicinity of the PAF system (Fig. 4) (Cliff et al. 1974; Exner 1976; Miller et al. 2011; Scharbert 1975; von Gosen 1989).

Based on paleomagnetic data, mapping, stratigraphy and sedimentological studies, Fodor et al. (1998) provided a detailed structural framework and kinematic sequence for the Miocene–Pliocene evolution of the Slovenian part of the Periadriatic fault, just east of our study area. They differentiated an Early Miocene compression, a Karpatian transtension and a Middle Miocene-to-Quaternary compressional event. They also found a complex pattern of block rotations in the vicinity of the PAF.

### Tonale fault

**Fig. 5:**
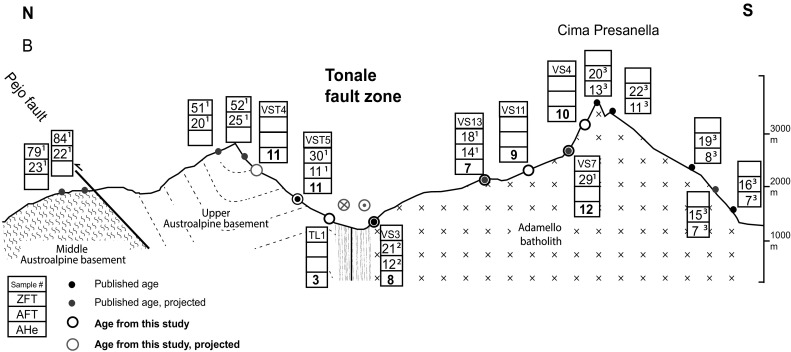
N–S cross section of the Tonale fault. Published ages are from ^1^Viola et al. (2003); ^2^Stipp et al. (2004); ^3^Reverman et al. (2012). Cross section modified after Dal Piaz et al. (2007). Trace of the section (*B*) is indicated in Fig. 3

The Tonale fault is the central-eastern segment of the PAF system (Figs. 3, 5). Its Cenozoic kinematic history is complex and records competing effects of terminal oblique convergence and rotation of the Adriatic indenter and simultaneous orogen-parallel extension (Mancktelow 1992). The western sector of the Tonale fault, adjacent to the Bergell intrusion, is primarily a greenschist-facies mylonite zone separating the Southern Alps from the amphibolite facies units of the Central Alps (Lepontine dome), which were exhumed to shallow crustal levels till the Pliocene (e.g., Campani et al. 2010; Mahéo et al. 2013). The eastern sector, instead, runs north of the Adamello complex and separates it from the Austroalpine units, where the alpine overprint is confined to greenschist shear zones and cooling to temperatures ≥300 °C is Variscan (e.g., Viola et al. 2003). In this sector only 5 km of estimated north side up vertical displacement occurred during dextral strike-slip movement between 32 and 30 Ma (Schmid et al. 1996). Pure dextral strike-slip movement continued until ~20 Ma, after which the mylonites of the eastern Tonale fault were offset by sinistral shearing presumably due to activity along the Giudicarie fault (Stipp et al. 2002).

### Giudicarie belt

The transpressive Giudicarie belt is a broad region of ESE-vergent thrusts and N–S trending sinistral strike-slip faults, bounded to the east by the sinistral Trento-Cles line and to the west by the sinistral transpressive Giudicarie line (Fig. 3), which are partially or totally the result of reactivation of Permian-Mesozoic normal faults (Castellarin et al. 1993; Picotti et al. 1995; Prosser 1998). The Giudicarie region was mainly affected by two phases of deformation during the Neogene: a Mid-Miocene phase, typified by the Valsugana thrust discussed below, and a younger, Late Miocene–Pliocene event, possibly associated with the initiation of the Montello–Friuli belt to the southeast (Caputo and Poli 2010; Castellarin and Cantelli 2000; Castellarin et al. 1992; Martin et al. 1998; Massironi et al. 2006; Viola et al. 2001).

The NNE-SSW-trending Giudicarie structural belt is oblique to the strike of the Southern Alps (Figs. 1, 3). Along this belt there is evidence of a structural boundary at crustal scale (Spada et al. 2013), whose surface expressions are major differences between the sectors east and west of the Giudicarie belt. West of the Giudicarie belt south-vergent structures are sealed by the Early Oligocene Adamello complex (Figs. 1, 3) (Brack 1981), whereas to the east there is little pre-Adamello deformation (Bigi et al. 1990) limited to the Eocene Dinaric deformation from the Dolomites eastwards (Doglioni and Bosellini 1987). During the Miocene the south-vergent structures, both west and east of the Giudicarie belt, propagated into the Po Plain foreland. Subsequently, the deformed Miocene sediments to the west were partially or completely eroded or buried beneath younger sediments (Pieri and Groppi 1981) and the internal parts of the fold and thrust belt have undergone little to no recent deformation (D’Adda et al. 2011; Wolff et al. 2012). To the east, instead, the foreland sediments continued to be involved in the deformation throughout the Pliocene–Pleistocene (Venzo 1977; Massari et al. 1986). At present, the direction of maximum horizontal compressive stress derived from focal mechanism is roughly perpendicular to the thrust fronts along the Giudicarie belt compatible with its dextral strike-slip reactivation (Vigano et al. 2008).

### Valsugana and Val Trompia thrusts

Two of the main structures of the Southern Alps are the Valsugana and Val Trompia thrusts that uplifted and exposed crystalline basement (Castellarin et al. 1988, 1993, Picotti et al. 1995) (Fig. 3). The timing of the deformation is well established along the Valsugana thrust, where AFT ages from the hanging wall of the thrust and detrital AFT data from the preserved syntectonic basin lying directly to the south constrain a period of intense activity between 12 and 8 Ma (Zattin et al. 2003, 2006). Clasts of Permian intrusives from the hanging wall appear in basin deposits by the Messinian, further arguing for intense uplift and erosion along the structure during the Late Miocene (Zattin et al. 2003).

The Val Trompia thrust borders the Adamello complex to the south, and it terminates to the east against the Giudicarie belt (Picotti et al. 1995) (Figs. 1, 3). It is part of a fold and thrust belt where AHe ages in both the footwall and hanging wall are within 1*σ* error indicating the fault has been inactive since the Mid-Miocene (Reverman et al. 2012).

## Thermochronological methods

The methodology is briefly outlined here; details are given in a supplementary file. Fission tracks in apatite (AFT) are damage zones in the crystal lattice formed during the radioactive spontaneous fission of ^238^U. At temperatures above ca. 110 °C tracks are annealed, whereas at temperatures below 60 °C tracks are retained. The range between these two temperatures is called the partial annealing zone (Carlson et al. 1999; Naeser 1979). AHe geochronology is based on the ingrowth of α-particles produced during the decay of U, Th and Sm. At temperatures exceeding 80 °C He is rapidly diffused and lost from the system, while at temperatures below 40 °C He is quantitatively retained (House et al. 2002; Wolf et al. 1996). The cooling rate, radiation damage and grain size control the temperature range for helium retention (Reiners and Farley 2001; Shuster and Farley 2009). AHe ages are corrected for He loss generated by α-ejection using a geometric correction factor (Ft). The total analytical error was computed as the relative standard error of weighted uncertainties on U, Th, Sm and He measurements. Ft corrections were made following Farley (2002).

The uncertainty of the age of a single-grain aliquot was calculated by the Gaussian error propagation from the U, Th, Sm and He measurements and from the estimated uncertainty of the Ft. The sample average is the unweighted arithmetic mean of the aliquot ages; the error is given as 2*σ* in the text and in Figs. 2 and 3. The scatter of the single-aliquot apparent ages derives mostly from submicroscopic inclusions, zoning of the alpha-emitting elements and from the differences between the sizes (diffusion domains) of the crystals (see, e.g., Fitzgerald et al. 2006). AHe analyses of the Austroalpine Karawanken samples were carried out in the Thermochronology Laboratory at Geoscience Center, University of Göttingen, Germany. The Southalpine samples were analyzed at the Noble Gas Lab, ETH Zürich.

## Thermochronology results

Table 1 presents AHe results from the Karawanken Mountains, Table 2 the new AFT data and Table 3 AHe data from the Southern Alps. AHe ages range from 2.9 to 28.4 Ma, with a majority of samples falling between 6 and 12 Ma. AFT ages from the Southern Alps range from 7.9 to 29.7 Ma. The low number of spontaneous tracks in our samples prohibited the systematic measurement of confined track lengths, necessary for a detailed assessment of the thermal history. Below we discuss the ages in the context of each of the main structural features associated with the sampling. Errors are given as 2*σ*.

**Table 1:** (U–Th–Sm)/He analytical data from the eastern Periadriatic fault

Sample	Grain ID	Lithology	Location			He		U238			Th232			Th/U ratio	Sm			Ejection correct. (Ft)	Uncorr. He age (Ma)	Ft-Corr. He age (Ma)	2*σ *(Ma)	Sample unweighted aver. ± 1*σ* (Ma)	
			Lat (°N)	Long (°E)	Altitude (m)	Vol. (ncc)	1*σ*	Mass (ng)	1*σ*	Conc. (ppm)	Mass (ng)	1*σ*	Conc. (ppm)		Mass (ng)	1*σ*	Conc. (ppm)						
KA10	a1	Hbl-granite	46°28.480′	14°35.623′	581	0.078	2.6	0.058	2.1	40.8	0.143	2.5	100.0	2.45	0.498	6	347	0.66	6.7	10.1	1.2		
	a2					0.020	4.7	0.023	3.2	16.8	0.068	2.5	50.4	3.00	0.310	7	230	0.79	4.0	5.1	0.6		
	a3					0.034	3.3	0.031	2.7	19.2	0.053	2.6	32.3	1.68	0.379	7	231	0.64	6.1	9.5	1.3	8.2	2.7
KA16	a1	Tonalite	46°32.680′	13°50.737′	653	0.177	1.6	0.094	1.9	65.8	0.079	2.5	55.0	0.84	0.241	7	169	0.63	12.8	20.5	2.5		
	a2					0.209	1.5	0.105	1.9	51.2	0.128	2.5	62.0	1.21	0.398	7	194	0.61	12.5	20.3	2.5	20.4	0.1
KA17	a1	Granodiorite	46°28.492′	14°37.341′	655	0.023	4.1	0.024	3.2	17.3	0.054	2.5	39.3	2.27	0.202	8	147	0.65	5.0	7.7	1.1		
	a2					0.041	3.4	0.038	2.4	18.2	0.121	2.5	57.2	3.14	0.374	7	177	0.72	4.9	6.8	0.8		
	a3					0.092	2.4	0.101	1.9	39.8	0.115	2.5	45.1	1.13	0.548	7	215	0.77	5.8	7.4	0.7	7.3	0.4
KA18	a1	Diorite	46°28.621′	14°36.452′	619	0.063	2.6	0.070	2.0	31.7	0.159	2.4	72.0	2.27	0.905	6	410	0.67	4.6	6.8	0.8		
	a2					0.029	4.0	0.042	2.3	32.7	0.044	2.6	33.7	1.03	0.361	7	278	0.73	4.3	5.9	0.7		
	a3					0.078	2.4	0.081	2.0	33.9	0.146	2.5	61.3	1.81	0.847	7	356	0.63	5.3	8.4	1.1	7.1	1.3
KA19	a1	Gabbro	46°28.553′	14°35.318′	605	0.165	1.7	0.064	2.0	13.2	0.354	2.4	73.0	5.52	0.505	7	104	0.78	9.0	11.5	0.9		
	a2					0.220	1.8	0.087	1.9	24.6	0.369	2.4	104.0	4.23	0.441	7	124	0.82	10.2	12.4	0.9		
	a3					0.245	1.6	0.119	1.9	17.6	0.478	2.4	71.1	4.03	0.887	6	132	0.82	8.5	10.3	0.7	11.4	1.1
KA27	a1	Metabasite	46°28.857′	14°38.605′	706	0.007	7.7	0.005	14.5	4.3	0.029	2.7	25.4	5.96	0.292	8	254	0.54	3.9	7.3	1.7		
	a2					0.028	3.8	0.017	4.1	2.7	0.088	2.5	13.9	5.19	1.552	6	246	0.72	4.6	6.3	0.8		
	a3					0.014	5.5	0.011	6.5	3.4	0.046	2.6	14.1	4.09	0.890	6	270	0.74	3.9	5.3	0.8	6.3	1.0
KA7	a1	Tonalitegneis	46°27.967′	14°36.967′	645	0.162	1.8	0.110	1.9	18.7	0.219	2.4	37.2	1.99	0.999	6	169	0.77	7.9	10.2	0.8		
	a2					0.115	2.1	0.105	1.9	22.9	0.204	2.4	44.6	1.95	0.736	6	161	0.74	6.0	8.1	0.8		
	a3					0.170	1.7	0.129	1.9	21.7	0.236	2.4	39.6	1.83	1.046	6	175	0.75	7.3	9.6	0.8	9.3	1.1
KS14	a3	Tonalite	46°25.954′	14°58.373′	982	0.079	2.4	0.075	2.0	20.3	0.148	2.5	39.9	1.97	0.637	7	172	0.76	5.7	7.5	0.7		
	a4					0.070	2.4	0.089	1.9	33.0	0.059	2.5	21.7	0.66	0.454	7	168	0.66	5.4	8.2	1.0		
	a5					0.147	1.9	0.135	1.9	23.4	0.209	2.4	36.1	1.54	0.974	6	169	0.76	6.3	8.4	0.7	8.0	0.5
KS-11	a1	Tonalite	46°27.055′	14°49.973′	708	0.084	5.3	0.149	1.9	30.3	0.245	2.4	49.80	1.64	0.834	8.4	169.85	0.69	3.25	4.7	0.68		
	a2					0.152	2.7	0.154	1.9	21.4	0.241	2.4	33.48	1.57	1.040	8.4	144.65	0.76	5.73	7.5	0.71		
	a3					0.079	3.1	0.096	1.9	27.4	0.131	2.5	37.45	1.36	0.532	8.4	151.67	0.72	4.98	6.9	0.75	6.4	1.5
KAR-25	a2	Tonalite	N46° 35.799′	E14° 08.624′	800	0.138	2.9	0.039	2.4	5.5	0.026	2.7	3.7	0.68	0.518	8	73	0.80	23.1	28.9	2.7		
	a3					0.026	6.3	0.008	7.7	4.777	0.0	2.8	13.0	2.715	0.2	8.5	101.0	0.56	14.6	26.274	5.3	27.6	1.8

**Table 2:** AFT analytical data from the Southern Alps

Sample number	Long. (°E)	Lat. (°N)	Altitude (m)	No. of crystals	Spontaneous		Induced		Dosimeter		U (ppm)	*P*(*χ*)^2^	Age (Ma) ± 2*σ*
					*N*_s_	*ρ*_s_	*N*_i_	*ρ*_i_	*N*_d_	*ρ*_d_			
VT2-10	10.3664	45.8446	2000	8	45	10.3	630	144	3658	114	16	64	14.5 ± 4.6
PBFT6	10.2185	45.8954	240	20	124	4.87	2211	86.8	3658	111	10	46	11.1 ± 2.2
PBFT8	10.1669	45.8820	280	20	98	10.1	1027	106	2859	110	12	84	18.9 ± 4.0
PBFT9	10.1659	45.8820	270	20	45	4.57	655	63.7	2859	107	7.4	92	13.8 ± 4.2
PBFT10	10.2810	45.9400	300	20	43	4.63	981	106	3658	102	13	53	7.9 ± 2.4
PBFT11	10.4157	45.8115	1990	20	91	9.09	574	57.4	2859	104	6.9	39	29.7 ± 7.0
PBFT12	10.6045	45.9473	600	20	68	7.68	1227	137	3658	110	16	3	11.3 ± 3.4
PBFT13	10.6045	45.9473	730	20	173	16.1	3476	323	3658	107	38	3.5	10.1 ± 2.2
BG72.2	10.4686	45.8185	640	20	57	4.65	806	65.8	3904	14.82	3.3	99	17.7 ± 4.9
VT1-10*	10.3783	45.8364	1935	20	177	26.4	3194	47.7	4454	143	40.7	86.9	9.9 ± 3.5
CDA1**	11.6051	46.1701	2130	19	58	8	1212	166	4910	103	20.6	97.8	9.1 ± 2.4
FRI**	11.2160	45.9677	1089	23	325	9.3	366	105	5924	125	5.1	39.4	202.5 ± 33.8

*N*_s_: number of spontaneous tracks counted on internal mineral surface; *ρ*_s_: spontaneous track density (×10^4^ cm^−2^); *N*_i_: number of induced tracks counted on external mica detector; *ρ*_i_: induced track density (×10^4^ cm^−2^); *N*_d_: number of dosimeter tracks counted on external mica detector; *ρ*_d_: dosimeter track density (×10^4^ cm^−2^); *P*(*χ*)^2^: probability of obtaining Chi-square value for *n* degrees of freedom (where *n* = number of crystals − 1); a probability >5 % is indicative of a homogenous population. AFT ages were obtained using the standard external detector method and the zeta calibration approach. The zeta value, obtained on Durango and Fish Canyon apatite standards (Hurford and Green 1983) for D. Seward is 360 ± 5, for R. Reverman (*) 257 ± 14, for M. Zattin (**) 346 ± 18. Ages given in this table and the text are central ages. Error is quoted as 2*σ*

**Table 3:** (U–Th–Sm)/He analytical data from the Southern Alps

Sample	Grain ID	Lithology	Long (°E)	Lat (°N)	Altitude (m)	Mass (μg)	4He (fmol)	238U (fmol)	232Th (fmol)	147Sm (fmol)	eU (ppm)	Uncorr. He age (Ma)	Analytical error (Ma)	Ejection correct. (Ft)	Ft-Corr. He age (Ma)	Mean age (Ma)	*σ* (Ma)
*Tonale fault (S)*																	
VS13	a1	Tonalite, Presanella unit	10.6556	46.2556	1935	2.86	2.13	298.49	359.44	467.18	35.18	4.30	0.06	0.71	6.03	6.7	0.6
	a2					1.87	2.35	313.08	361.35	429.38	55.78	4.57	0.07	0.68	6.72		
	a3					2.29	1.97	239.57	298.48	421.77	35.67	4.90	0.08	0.68	7.24		
VS4	a2	Tonalite, Presanella unit	10.6393	46.2275	2916	1.52	2.03	178.64	289.13	297.62	43.71	6.37	0.11	0.65	9.86	9.8	0.1
	a3					2.11	2.51	215.98	313.81	431.67	36.58	6.69	0.10	0.69	9.67		
VS11	a3	Tonalite, Presanella unit	10.6565	46.2429	2300	1.42	1.53	183.76	195.84	227.66	42.12	5.14	0.10	0.65	7.89	8.5	1.1
	a2					5.11	6.56	669.20	819.63	1138.40	44.42	5.89	0.07	0.76	7.77		
	a1					2.23	3.34	363.68	81.04	233.24	42.04	6.75	0.09	0.70	9.71		
VS7	a3	Tonalite, Presanella unit	10.6431	46.2316	2703	4.61	5.83	414.27	716.61	903.06	34.14	7.73	0.09	0.74	10.45	11.6	1.2
	a2					4.73	4.81	320.32	277.10	474.96	20.99	9.64	0.11	0.75	12.86		
	a1					4.60	7.94	514.08	832.86	1024.97	41.51	8.65	0.10	0.75	11.60		
*Fault zone*																	
VS3	a1	Schist, Tonale unit	10.6553	46.2744	1362	5.31	0.26	13.04	145.73	74.24	2.82	4.36	0.21	0.76	5.72	8.3	3.8
	a2					3.89	0.39	44.26	74.13	134.25	4.27	4.93	0.17	0.75	6.56		
	a3					3.59	0.34	9.15	80.35	50.81	2.43	9.32	0.32	0.73	12.72		
DIM1	a1	Tonalite, Presanella unit	10.8650	46.3202	850	3.80	32.38	145.46	2756.35	475.00	48.67	32.05	1.15	0.71	45.09	6.4	0.8
	a2					2.24	6.55	768.46	1669.20	579.30	122.93	4.39	0.08	0.66	6.62		
	a3					2.68	7.69	842.78	1604.24	473.47	108.03	4.91	0.08	0.69	7.08		
	a4					2.24	2.62	418.89	540.82	231.68	58.02	3.73	0.07	0.67	5.58		
*Tonale fault (N)*																	
TL1	a1	Schist, Tonale unit	10.6677	46.2881	1376	15.97	6.90	1690.26	22.28	2251.55	25.49	3.13	0.04	0.83	3.76	2.9	0.8
	a2					46.16	6.46	2506.07	135.16	2795.57	13.25	1.96	0.02	0.88	2.24		
	a3					7.48	1.27	458.49	5.10	581.37	14.74	2.13	0.04	0.78	2.71		
VST4	a1	Schist, Tonale unit	10.6062	46.2960	2276	1.17	0.63	56.63	3.89	57.58	11.84	8.46	0.24	0.64	13.28	10.8	2.1
	a2					11.51	8.61	753.25	50.19	599.49	16.05	8.68	0.11	0.83	10.42		
	a3					10.48	0.53	43.77	b.d.	209.95	0.99	9.26	0.22	0.83	11.17		
	a4					1.61	0.20	27.61	b.d.	37.29	4.06	5.52	0.37	0.68	8.17		
VST 5	a1	Schist, Tonale unit	10.6375	46.2792	1624	5.30	10.98	951.52	173.88	1379.23	44.82	8.51	0.10	0.78	10.89	11.3	0.5
	a2					4.68	11.68	1039.44	149.75	1237.88	54.96	8.37	0.10	0.75	11.18		
	a3					6.20	15.60	1256.30	189.61	1501.70	50.22	9.24	0.11	0.78	11.82		
	a4																
*Giudicarie belt*																	
01RZ12	a1	Eocene, Ponte Pia Fm	10.9726	46.1307	1023	20.52	25.90	1532.75	3700.64	3234.05	27.75	8.35	0.09	0.84	9.94	10.4	0.6
	a2					5.98	7.11	472.32	719.57	773.48	25.51	8.58	0.12	0.77	11.16		
	a3					1.62	1.82	126.90	363.92	308.44	30.99	6.64	0.13	0.65	10.24		
02RZ12	a1	Eocene, Ponte Pia Fm	10.9761	46.1307	1023	4.73	4.85	298.03	773.99	750.73	24.00	7.82	0.11	0.75	10.43	9.9	0.5
	a2					6.03	7.14	454.71	1214.65	925.29	29.08	7.48	0.09	0.77	9.76		
	a3					4.60	5.14	361.08	861.52	757.27	29.01	7.06	0.10	0.75	9.44		
05RZ12	a1	Eocene, Ponte Pia Fm	11.0653	46.2723	332	9.57	33.44	1027.03	2469.96	952.81	39.79	16.17	0.18	0.79	20.51	17.0	3.3
	a2					10.62	31.38	1040.17	3448.75	1264.08	41.18	13.20	0.15	0.80	16.56		
	a3					4.63	9.71	407.81	1364.50	508.90	37.19	10.38	0.13	0.74	14.04		
vd 11	a1	Tonalite, Western Adamello unit	10.5644	46.0859	1908	3.16	4.50	418.12	689.39	737.49	43.62	6.00	0.08	0.72	8.35	7.9	0.6
	a2					2.40	2.51	291.39	449.86	513.95	39.31	4.89	0.08	0.67	7.27		
	a3					6.63	7.72	693.18	1150.45	1346.53	34.53	6.20	0.08	0.76	8.21		
VM5-10	a1	Tonalite, Western Adamello unit	10.4713	46.1278	2522	8.99	8.26	973.46	1569.69	1804.27	35.47	4.76	0.06	0.79	6.02	5.9	0.1
	a2					1.98	2.14	309.70	506.95	492.90	51.37	3.86	0.07	0.66	5.85		
	a3					2.86	3.09	422.43	672.11	652.42	48.22	4.12	0.07	0.71	5.85		
VM6-10	a1	Tonalite, Western Adamello unit	10.4420	46.1306	2070	7.01	7.82	863.04	90.33	813.56	30.21	6.82	0.10	0.77	8.80	7.8	1.0
	a2					3.91	5.13	201.54	270.14	231.38	16.13	14.99	0.31	0.73	20.62		
	a4					2.06	2.21	299.94	284.29	241.77	42.42	4.67	0.07	0.68	6.85		
	a5					4.37	5.36	584.63	586.77	498.40	39.43	5.75	0.08	0.76	7.60		
*Valsugana*																	
CDA1	a1	Cima D’Asta granite	11.6051	46.1701	2480	1.19	1.03	154.64	336.14	794.26	46.56	3.39	0.07	0.57	5.90	9.5	3.2
	a2					1.58	2.79	239.39	328.61	472.20	47.75	6.77	0.13	0.64	10.57		
	a3					0.92	1.03	76.34	182.81	442.09	30.86	6.61	0.19	0.55	11.99		
CAL1	a1	Cima D’Asta granite	11.4955	46.0876	722	4.54	4.86	574.34	858.45	1384.39	40.68	4.83	0.07	0.74	6.53	6.9	0.3
	a2					1.68	1.70	228.36	283.57	494.99	41.86	4.44	0.10	0.63	7.05		
	a3					1.35	1.35	184.12	193.63	356.27	40.67	4.52	0.11	0.64	7.07		
CAL11	a4	Paragneiss	11.2985	46.0514	1640	3.19	4.52	79.34	219.64	551.61	9.71	26.33	0.40	0.71	36.94	28.4	8.5
	a2					7.27	12.31	359.93	1090.09	2633.63	20.05	15.27	0.20	0.77	19.87		
	a1					4.04	7.08	158.26	464.34	1553.48	15.65	20.07	0.30	0.71	28.35		
CAL2	a5	Cima D’Asta granite	11.5009	46.0966	875	2.75	5.73	721.42	628.22	926.00	75.32	5.10	0.07	0.70	7.25	9.0	2.3
	a6					1.96	3.76	318.12	199.45	368.61	44.36	7.95	0.11	0.68	11.64		
	a4					2.01	4.88	575.03	572.45	904.85	84.23	5.32	0.12	0.66	8.04		
FRI	a1*	Paragneiss	11.2160	45.9677	1089	1.42	81.92	73.26	346.66	1010.41	25.64	392.46	5.90	0.58	678.15	27.2	4.5
	a2					1.78	6.28	141.14	413.33	1075.33	31.68	20.10	0.27	0.66	30.44		
	a3					1.11	2.45	71.31	259.30	794.13	28.19	14.04	0.26	0.58	24.01		
	a4*					1.56	11.66	86.05	324.98	1185.22	24.60	53.96	0.74	0.66	82.33		
*Val Trompia-Val Camonica*																	
PBFT6	a1	Permian, Auccia Volcanics	10.2185	45.8954	240	1.22	0.31	38.01	76.75	394.17	10.90	4.13	0.18	0.61	6.73	7.0	0.4
	a2					3.90	2.36	226.52	437.14	1408.81	20.04	5.46	0.07	0.75	7.30		
	a3*					1.68	0.79	37.15	104.41	497.14	8.71	9.55	0.23	0.66	14.37		
	a4*					1.75	2.79	91.50	83.53	227.10	15.16	19.31	0.27	0.69	28.01		

* Grain rejected from age calculation (see text for details)

### Karawanken Mountains

Samples for AHe analysis were taken from the gabbroic to granitic members of the Permian to Triassic North Karawanken plutonic and the Oligocene tonalitic southern belt along the eastern part of the PAF system and from the Paleozoic basement (Figs. 2b, 4; Table 1).

The *α*-ejection corrected mean AHe ages from within the positive flower structure of the Karawanken vary from 11 to 6 (±2) Ma without any systematic trends (Table 1). The most obvious change occurs outside the flower structure where AHe ages are substantially older. A site along the PAF but west of the flower structure and HMV fault yields an AHe age of 20 Ma (KA16) (Fig. 2b). A further AHe age of 28 ± 4 Ma (KAR25) was derived for the Oligocene Reifnitz tonalite north of the Karawanken Mountains, which is located in the basement of the Neogene Klagenfurt basin, in the footwall of the North Karawanken unit (Fig. 2b).

### Tonale fault

Samples across the Tonale fault were taken as close as possible to previous AFT and ZFT samples but only a few locations provided suitable apatites for AHe dating. AHe ages across the Tonale fault record mostly Late Miocene cooling, though discrepancies occur (Figs. 3, 5). AHe ages north of the line are ~11 Ma (VST4 and VST5), while to the south ages at similar elevations show a larger spread between 6 and 12 Ma. Interestingly, ZFT analyses revealed a significant difference in cooling ages (Viola et al. 2003) with the oldest ages in the footwall of the Pejo fault (ca. 80 Ma), younger ages between the Pejo and Tonale faults (ca. 50–20 Ma) and youngest ages south of the Tonale fault (Fig. 3). In contrast, AFT and our new AHe ages from samples in the same area do not show differential cooling (Figs. 3, 5) but imply Miocene cooling as a coherent block across the Tonale fault. A Pliocene (2.9 Ma, TL1) AHe age is found within the Austroalpine basement north of the line (Figs. 3, 5). Given that all other samples in the immediate area yield Miocene ages, we interpret this sample to have been recently reset (i.e., due to localized fluid flow), rather than indicating significant recent exhumation.

### Valsugana thrust

**Fig. 6:**
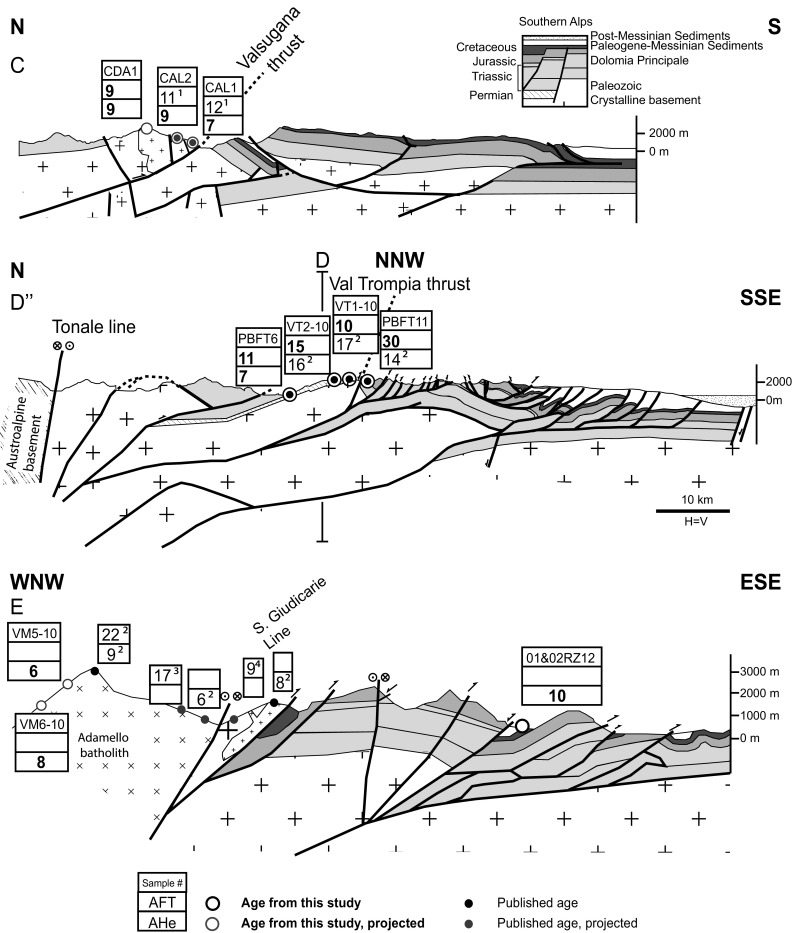
Cross sections of the central Southern Alps. *Section C*: N–S cross section of the Valsugana thrust. Published ages are from ^1^Zattin et al. (2003). Cross section modified after Caputo and Bosellini (1994), Selli (1998) and Barbieri and Grandesso (2007). *Section D* and *D*″: N–S and NNW–SSE cross section of the Val Trompia thrust. Published ages are from ^2^Reverman et al. (2012). Cross section modified after Schoenborn (1992) and Picotti et al. (1995). *Section E*: Ca. W–E cross section of the Giudicarie belt. Published ages are from ^2^Reverman et al. 2012; ^3^Viola 2000, ^4^Viola et al. 2001. Cross section after Picotti et al. 1995. Traces of the sections are indicated in Fig. 3

AHe ages were obtained from samples that were previously dated by AFT analysis from the hanging wall of the Valsugana thrust (Zattin et al. 2003) (Figs. 3, 6, Section C). The highest elevation sample (CDA1) has AHe and AFT ages that are essentially the same, indicating rapid cooling at ~11 Ma, in agreement with previous studies (Zattin et al. 2003, 2006). While AFT ages are invariant at lower elevations, the AHe ages get slightly younger (~7 Ma). Two AHe ages were obtained on samples from basement highs that were never buried by more than 4 km of sediments since the Cretaceous (Bosellini and Doglioni 1986) (Fig. 3). These samples (FRI and CAL11) yield Oligocene AHe (27 and 28 Ma) and Mesozoic AFT ages (77 and 202 Ma). In sample FRI two grains (a1 and a4) yield anomalously older ages and the remaining two grains yield Oligocene ages similar to those found in sample CAL11 (Table 1). Both samples have a high dispersion of individual grain ages, which suggests, along with the relatively large discrepancy with their AFT ages, very slow cooling since the Mesozoic.

### Val Trompia thrust

We present ten new AFT ages and one new AHe age from the Val Trompia area (Figs. 3, 6, Section D″/D). All ages record Middle to Late Miocene cooling. The AFT data show a discrepancy in ages across the fault, with 29.7 Ma (PBFT11) in the footwall and 14.5 Ma (VT2-10) and 9.9 Ma (VT1-10) in the hanging wall. However, no major discrepancies in the AHe ages occur across the fault, where the ages from the hanging wall are slightly older, but within error, of those in the footwall, 16.4 and 13.5 Ma, respectively (Reverman et al. 2012).

A noticeable problem in the hanging wall is that the AFT ages are younger than the AHe ages. Inversion of AFT and AHe ages has been identified in several studies and has yet to be fully explained, though it is often argued that the problem lies either in zonation of the parent nuclides, or He implantation from neighboring grains with high U content, or in an imperfect understanding of helium diffusion kinetics for samples with complex and slow cooling histories (Ault and Flowers 2012; Flowers et al. 2009; Green and Duddy 2006; Shuster et al. 2006; Spiegel et al. 2009), all of which are applicable to these samples.

### Giudicarie belt

In the Giudicarie belt samples were collected with the aim of quantifying vertical offsets across this major boundary. However, suitable apatite bearing lithologies are scarce. Three samples were taken from the Ponte Pià formation, reworked intermediate tuffs of the Eocene Giudicarie-Insubric Flysch (Castellarin et al. 1993, 2006; Sciunnach 1994) (Figs. 2, 6, Section E). The northern sample (05RZ12) records an AHe age of 17 Ma, while the two southern samples (01RZ12 and 02RZ12) yield ages of ~10 Ma.

## Discussion

In the following, we will first discuss evidence for Late Miocene exhumation along the eastern PAF segment in the Karawanken Mountains, then discuss late-stage cooling in the Southern Alps, followed by a synthesis of evidence for widespread latest Mid-Miocene to Late Miocene foci of deformation and exhumation. We will proceed with a discussion of the potential engine driving Late Miocene exhumation in the frame of the broader Neoalpine evolution and Adriatic indentation.

### Late-stage exhumation at the leading edge of the indenter in the Karawanken Mountains

Our new AHe cooling ages ranging from 13 to 4 Ma from the Karawanken flower structure in conjunction with the clastic record from the Klagenfurt basin (Nemes et al. 1997 and references therein) indicate a hitherto undated late Neogene exhumation pulse along the eastern segment of the PAF. This pulse was associated with surface uplift and topography growth during the formation of the flower structure and thrusting of the North Karawanken unit onto proximal clastic deposits of the Klagenfurt basin, the only flexural basin inside the Alps. The coarsening-upward sequence of the basin fill derived from the Karawanken clearly reveals the initiation of uplift and fast-growing relief of the chain (Polinski and Eisbacher 1992 and references therein). Further evidence for rapid cooling comes from an AFT age of 12.8 ± 1.8 (Nemes 1996) from the Karawanken tonalite (Fig. 2b). This age is only slightly older than the AHe ages presented herein, suggesting rapid upper crustal cooling and unroofing on the order of 3–5 km since latest Mid-Miocene time.

Importantly, AHe ages derived from outside the Karawanken flower structure are distinctly older: Along the western segment of the eastern PAF, no similar Late Neogene shortening occurred, as evidenced by an older AHe age of 20 Ma (KA16) from west of the Hochstuhl–Möll Valley fault system (Fig. 2b). Combining our new age with published AFT ages of 26 ± 2 Ma from the same locality and 25 ± 2 Ma further west (Hejl 1997) attests to an earlier phase of Late Oligocene to Early Miocene cooling. A further AHe age of 28 ± 4 Ma from the Reifnitz tonalite (KAR25) corroborates that Late Miocene cooling has not seriously affected the Austroalpine lid from the immediate surroundings of the Karawanken flower structure.

An earlier cooling phase may be related to Late Oligocene to earliest Miocene NNW-ward advancement of the eastern Adriatic indenter (Pomella et al. 2011, 2012), and possibly its incipient northward subduction (Handy et al. 2014; Lippitsch et al. 2003) leading to a phase of major extrusion, mainly in the Tauern Window. In its western part rapid exhumation (2–4 mm/a) was bracketed between 20 and 12–10 Ma (Fügenschuh et al. 1997). During this early extrusion phase strain concentration in front of the tip of the indenter within the Tauern Window was influenced primarily by the occurrence of wedge-shaped blocks of Austroalpine units at the leading edge of the eastern Adriatic indenter (Scharf et al. 2013). These blocks may have undergone internal fragmentation into the Drau–Möll and Rieserferner blocks (Fig. 2a) at the end of the Oligocene triggering ESE-ward migration of exhumation in the Eastern Tauern subdome (Favaro et al. 2015).

### Late-stage exhumation in the Southern Alps

Throughout most of the Southern Alps exhumation of deep crustal material did not occur and erosion of less than 10 km is evident from the Mesozoic ZFT ages and the Mesozoic-to-Eocene AFT ages recorded in the Orobic Alps (Bertotti et al. 1999; Zanchetta et al. 2011, 2015), the Giudicarie region (Zattin et al. 2006) and the Dolomites (Emmerich et al. 2005). Shortening during Alpine orogeny led to significant exhumation mainly in localized areas in and around the Giudicarie belt. We find Late Miocene ages (AFT and AHe) as a first-order feature of the cooling and exhumation pattern of the central-eastern Southern Alps. Major Late Miocene exhumation along the Valsugana thrust, already indicated by Tortonian AFT ages from the Valsugana thrust (Zattin et al. 2006) and its southern foreland (Monegato and Stefani 2010), is corroborated by our new AHe data. Unroofing of ca. 4 km of sediment along the thrust and exposure of the crystalline basement correlates with rapid growth, migration and erosion of the Southalpine fold-and-thrust belt. AFT ages to the north and immediately to the west of the Valsugana thrust (e.g., CAL11 77.4 Ma, Fig. 2) are Mesozoic (Emmerich et al. 2005; Zattin et al. 2003) indicating that Miocene exhumation was limited to and focused along the fault. Moreover, the Oligocene AHe ages in the western sector of the Valsugana thrust indicate differential amounts of exhumation along this structure, which cannot be explained simply by differential thickness of Mesozoic sediments due to inherited structural highs and lows (Bosellini and Doglioni 1986). This pattern of focused exhumation can be extended to the Val Trompia fault, where Middle to Late Miocene AFT ages are found juxtaposed with Oligocene ages across the fault (Fig. 6, Section D″/D).

The Late Miocene ages found in the Giudicarie belt and Adamello area are ascribed to uplift associated with basement ramps that folded the earlier/older thrust sheets in the area (Fig. 6, Section D″/D) during thickening of the wedge. The uplift of the entire Adamello complex likely occurred due to it acting as a rigid block, whereas elsewhere in the Southern Alps an inherited network of Mesozoic faults allowed for strain partitioning and localization of deformation. A similar Tortonian pulse of exhumation has also been reported for the northern sectors of the central Southern Alps based on AFT data (Zanchetta et al. 2015).

Along the Giudicarie belt, the Miocene AHe ages from Eocene sedimentary units (Ponte Pia Fm) indicate that locally significant erosion affected not only the deepest units of the Southern Alps along the main thrusts but locally also the top of the sedimentary pile with removal of up to 2 km of overburden between deposition and the Mid- to Late Miocene, when exhumation occurred. The overburden of the Eocene units located in the core of the Giudicarie belt could have been partly due to thrusting (samples 01RZ12 and 02RZ12) as suggested by their location between imbricated thrust sheets and their AHe age of 10 Ma (Fig. 6, Section E). Nevertheless for the sample from the Eocene units with an AHe age of 17 Ma (05RZ12) located to the east of the Trento-Cles fault, there is no unequivocal evidence of overthrusting suggesting that the nature of the overburden there could have been sedimentary. The 2-km estimate of sedimentary overburden is well in line with vitrinite reflectance and solid bitumen reflectance measurements from the Dolomites indicating about 1800 ± 200 m of eroded thickness (Grobe et al. 2015) as well as the preservation of Oligocene/Miocene coastal sediments at ca. 2600 m altitude in the Eastern Dolomites (Keim and Stingl 2000). Moreover, the location of the older sample supports the argument for a general southward advance of deformation for the eastern Southern Alps (Mellere et al. 2000; Zattin et al. 2006). This argument is based on the structural evolution of the belt and of its foreland basin. In fact, an increase in subsidence in the Venetian Friuli basin is also recorded at 17 Ma (Mellere et al. 2000), while maximum subsidence occurred from the Serravallian to the Messinian in relation to tectonic loading due to the advancing Southalpine thrust belt. Given the petrologic evolution upsection of the foreland basin sediments, from carbonates (Cretaceous-Jurassic), to dolomites (Triassic), to metamorphic and volcanics (Permian), rapid progressive unroofing of the Southalpine basement must have occurred during the Serravallian to Messinian (Massari et al. 1986; Mellere et al. 2000; Zattin et al. 2003). The timing of basin subsidence and aggradation fits well with our results about the overall timing of exhumation in the Southern Alps and suggested geodynamic models (Bressan et al. 1998; Massironi et al. 2006).

In summary, major S-directed thrusting and rapid growth of the Southalpine fold-and-thrust belt within the eastern Adriatic indenter was active during the Serravallian (Castellarin and Cantelli 2000) or the Tortonian (e.g., Doglioni and Bosellini 1987; Schoenborn 1992), corroborated by thermochronological data evidencing Mid-Late Miocene exhumation. Accordingly, the eastern Adriatic indenter must have behaved in a rather rigid manner for ca. 8 myr with only little internal deformation in its northernmost sector (Caputo and Poli 2010 and references therein) after initial indentation at ca. 21 Ma. In “Driving forces for Late Miocene deformation and exhumation” section we will discuss in more detail how and why strain was accommodated differently in response to indentation.

### Further evidence for late-stage exhumation

Our new thermochronological data from the Southern Alps and the Karawanken Mountains clearly indicate that latest Middle to Late Miocene unroofing is a widespread phenomenon at the leading and the western edge as well as within the eastern Adriatic indenter. Further evidence for NNW–SSE shortening and related exhumation exists at the northeastern edge of the indenter: east of the Lavant fault vitrinite reflectance and AFT data (10–14 Ma) from the northern Sava fold (Fig. 1) (Boc anticline) indicate Late Miocene shortening, basin inversion and exhumation on the order of 2.5–3 km south of the PAF (Sachsenhofer et al. 2001; Tomljenović and Csontos 2001). Folded latest Early and Middle Miocene rocks east of the Lavant fault also suggest that pronounced dextral transpression and deformation occurred during the Late Miocene (Fodor et al. 1998). According to these authors, dextral transpression along the Slovenian part of the PAF system culminated during the Late Miocene, in line with the evolution of the Karawanken flower structure. Evidence for yet younger, Plio-Pleistocene activity of the PAF system and the Sava fault associated with uplift of the Slovenian Kamnik Mountains has recently been shown in a study on cave sediments (Häuselmann et al. 2015). This might indicate a migration of Late Miocene exhumation from the North Karawanken Mts. southward into the Kamnik Mts. during Pliocene times during ongoing or renewed transpression.

As outlined above (“Late-stage exhumation at the leading edge of the indenter in the Karawanken Mountains” section) the push of the eastern Adriatic indenter initiated rapid exhumation within the Tauern Window in the Early Miocene (Foeken et al. 2007; Fügenschuh et al. 1997; Scharf et al. 2013). Processes related to indentation persisted throughout the Miocene and may be ongoing today (e.g., Caporali et al. 2013; Massironi et al. 2006), but their rates slowed down during Middle to Late Miocene time (e.g., Fügenschuh et al. 1997; Schneider et al. 2015). Based on thermochronology data from tunnel and surface samples from the Penninic Hochalm-Ankogel dome, that yielded slightly older AFT and AHe ages than those reported here (Figs. 2, 4), Foeken et al. (2007) suggested that the present-day topography evolved between 10 and 7 Ma during a phase of slow cooling (2–4 °C/Ma), after the major exhumation phase (ca. 22-16 Ma) associated with rapid cooling (40 °C/Ma). This Late Miocene phase of relief evolution in the SE Tauern Window is in line with palinspastic restorations that denote a Late Miocene age to the evolution of the Eastern Alps topography into a more mountainous landscape and a switch from a N–S-directed to a W–E-directed drainage system (Brügel et al. 2003; Frisch et al. 1998; Robl et al. 2008).

The Hochstuhl–Möll Valley fault system delimits the North Karawanken block to the west and shortening by thrusting disappears within a few kilometers, as also evidenced by narrowing of the westernmost Klagenfurt basin. W of the Hochstuhl fault, the basin fill is composed of about 150 m of gently folded Neogene strata, which die out laterally after a few kilometers, as well as a few tens of meters of Quaternary deposits. In contrast, to the east of the fault more than 600 m of flat-lying Tertiary clastics are overlain by up to 150 m of Quaternary deposits (Polinski and Eisbacher 1992). No flower structure nor thrust fault is known further west along the Pusteria–Gailtal fault though a subvertical ductile strike-slip duplex (Eder unit) with Early Oligocene cooling ages has been reported from the Carnian Alps (Läufer et al. 1997).

**Fig. 7:**
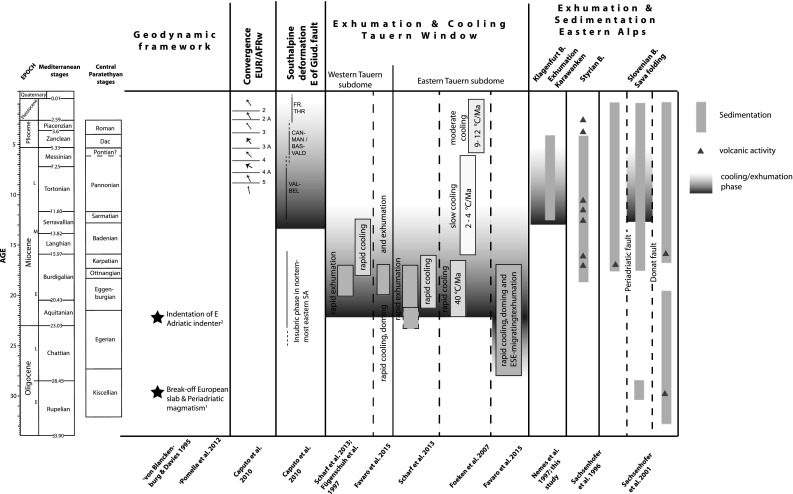
Summary of episodes of major deformation, cooling, exhumation as well as sedimentation and volcanic activity for the Eastern and Southern Alps. Chronostratigraphic correlation chart for the Mediterranean and Paratethyan stages according to Piller et al. (2007). Southalpine thrust systems: *VAL-BEL* Valsugana and Belluno, *CAN-MAN* Cansiglio-Maniago, *BAS-VALD* Bassano-Valdobbiadene, *FR.* *THR* frontal thrusts

The young ages reported here contrast with Paleogene AHe ages from Austroalpine units east of the Tauern Window (Wölfler et al. 2011) (Fig. 2a). There, hilly and moderately shaped remnants of Miocene planation surfaces are widespread (“cold spots” according to Hejl 1997) as opposed to the steep relief, at places in excess of 1500 m of the Karawanken Mts. and the Tauern window, without such paleosurfaces. This part of the Eastern Alps has experienced significantly less postcollisional shortening and exhumation and no upright folding in contrast to the Tauern Window with high-amplitude folding, major exhumation and erosion (e.g., Rosenberg et al. 2015). Our AHe data in conjunction with published age constraints allow one to construct an improved pattern of spatial heterogeneity of exhumation of the Austroalpine unit, which is more complex than previously thought. Distinct blocks in the Eastern Alps and northernmost Dinarides experienced a Late Miocene widely distributed deformation phase accompanied by significant rock uplift (Fig. 7).

Wrench deformation along the eastern PAF system reveals a peculiar pattern of strain localization/concentration: Strain was accommodated over a wide area in the SE Alps and NE Dinarides between Medvenica Mts./Karlovac basin (van Gelder et al. 2015) and the PAF system south of the Pohorje Mts. (Sava folds) (Fig. 1). In contrast, in the Karawanken Mountains, strain localization was most efficient and strain was concentrated within the narrow belt of the flower structure, with mainly NW thrusting and formation of the flexural Klagenfurt basin (Nemes et al. 1997).

We attribute different deformation styles to contrasting preceeding thermal evolution and resulting lateral differences in crustal strengths: Pervasive Sava folding resulted from thermal crustal weakening due to addition of heat during syncollisional Early Miocene magmatism and extreme extension in the Early-Middle Miocene (Sachsenhofer et al. 2001; Fodor et al. 2008 and references therein) (Fig. 7). Weak lithosphere has also been reported for the Tauern Window (Genser et al. 2007; Willingshofer and Cloetingh 2003 and references therein), where northward movement of the eastern Adriatic indenter was initially accommodated by major ductile folding, exhumation and erosion as well as lateral extrusion. In contrast, such folding did not occur in the Karawanken Mountains and in the Southern Alps. Here mechanically stronger crust, which had experienced only weak Alpine metamorphic overprint, may have promoted localized strain concentration along faults (Karawanken flower structure; Valsugana thrust) instead.

### Driving forces for Late Miocene deformation and exhumation

Based on our new low-temperature thermochronology ages and published data we observe a shift of strain distribution during the Miocene. Early Miocene exhumation is clearly focused within the Tauern Window in front of the Eastern Adriatic indenter and the wedge-shaped Austroalpine blocks (Rieserferner and Drau-Möll blocks in Fig. 2a) (e.g., Fügenschuh et al. 1997; Scharf et al. 2013) (Fig. 7). Late Miocene exhumation is, however, more distributed, but also of smaller magnitude, indicating that convergence between the eastern Adriatic indenter and Europe is less focused and thus accommodated differently. Based on brittle fault analyses yielding predominant extensional and strike-slip states of stress in the Tauern Window, Bertrand et al. (2015) argued for such a switch in accommodation mechanism with initial vertical extension followed by strike-slip and normal faulting under constant N–S compression. This shift was triggered after orogen-scale folds were nearly isoclinal and the push of the indenter needed to be accommodated differently, leading to normal faulting and E–W extension within the Tauern Window. Importantly, this second stage initiated in the Late Miocene (Bertrand et al. 2015), coeval with more widespread exhumation in the Southern and Eastern Alps outlined above.

A possible mechanism leading to the observed exhumation pattern may lie in a shift of the mechanical coupling state and rheological behavior between the orogenic wedge and adjacent plates. Based on lithosphere-scale analogue modeling, Willingshofer and Sokoutis (2009) demonstrated that coupling is a key process for stress transmission and thus for the resulting deformation pattern. The amount of coupling will increase during continental collision following subduction. They correlated their experimental results with the pattern of deformation in the Eastern Alps and observe a switch from north-directed to south-directed thrusting as well as initiation of internal deformation of the indenter between ca. 12 and 10 Ma. Based on numerical modeling Robl and Stüwe (2005) argue for a strong decrease in rheology contrast between the initially much stiffer Adriatic indenter into the softer European margin. Interestingly, substantial strengthening of the indented Eastern Alpine orogenic wedge occurred since ca. 13 Ma (Robl and Stüwe 2005). These modeling results are well in line with our findings of a latest Mid- to Late Miocene shift from focused exhumation outside of the indenter (mainly Tauern Window) to more widespread exhumation within and along the rims of the indenter (Fig. 7). Major coupling of the Alpine wedge and the South Alpine fold and thrust belt is also evidenced by the fault framework and fault linkages between the Austroalpine and Southalpine domains, which were fully developed during the latest Miocene–early Pliocene (Bartel et al. 2014a, b; Massironi et al. 2006).

A further plausible mechanism may have been a reorganization of the stress field leading to a change in boundary conditions operating on the orogen. Neogene convergence rates based on shortening values are 0.6–1 cm/year (Rosenberg and Berger 2009). However, magnetic anomalies and structural analyses indicate a change in relative plate motion of Africa with respect to Europe from NE to NNW 16 Ma ago and from NNW to NW 8.5 Ma ago (Caputo and Poli 2010 and references therein). Such rotations may have governed the compressional stress field in the study area and may have intensified strain transfer and exhumation.

In summary, we suggest that a major change of the coupling state of the orogen, possibly enhanced by a reorganization of the large-scale stress field between Africa and Europe initiated widespread latest Mid- to Late Miocene deformation, thrusting and exhumation at various sites within, at the rim and at the tip of the indenter.

### Young uplift in the Eastern Alps?

Triggered by the detection of a dramatic increase in sediment flux to circum-Alpine basins since ca. 5 Ma (Kuhlemann et al. 2002) a large number of studies focused on the question, whether there has been a coeval, possibly climatically triggered increase in exhumation and erosion (e.g., Cederbom et al. 2004; Willett et al. 2006). In situ as well as detrital thermochronological data challenge sediment budget calculations and instead imply earlier phases of rapid exhumation or long-term steady-state conditions for the Western and Central Alps (e.g., Bernet et al. 2009; Glotzbach et al. 2011; Fox et al. 2015). Only few studies have addressed this point for the Eastern Alps (Wölfler et al. 2012), which are tectonically more active than the Western and Central Alps, where convergence stopped at ca. 6 Ma (Battaglia et al. 2004; Grenerczy et al. 2005). In the Eastern Alps, an increasing body of data is testifying to a yet younger stage of uplift and/or erosion (e.g., Wagner et al. 2010). Genser et al. (2007) report late-stage surface uplift of the eastern Molasse basin on the order of 400 m since ca. 6 Ma. Gusterhuber et al. (2012) find evidence for even more intense erosion of 1–2 km of the Alpine foreland basin since Late Miocene times. Pliocene-Quaternary uplift also occurred in the Styrian basin (Sachsenhofer et al. 1997), the western part of the Pannonian basin (Bada et al. 2001) and the easternmost unglaciated part of the Eastern Alps (Legrain et al. 2014, 2015; Wagner et al. 2010) (Figs. 1, 2). The cause of this uplift is controversially debated, being associated with either (1) crustal delamination and/or convective removal of thickened lithosphere (e.g., Genser et al. 2007), (2) the coeval major reorganization of the external stress field in the ALCAPA region (e.g., Horváth and Cloetingh 1996; Peresson and Decker 1997). Our new AHe data with the majority of ages ranging from 11 to 6 Ma clearly corroborate that not enough tectonic and/or erosional exhumation has occurred since then to be recorded by low-temperature thermochronology. Thus, our findings do not support an orogen-wide drastic increase in denudation during Pliocene and Pleistocene times.

## Conclusions

- AHe data from the eastern Periadriatic fault reveal a Late Miocene phase of activity and exhumation leading to the formation of the Karawanken flower structure and the infill of the Klagenfurt basin.
- New thermochronological data from the central-eastern Southern Alps constrain a coeval phase of uplift and erosion during the latest Mid- to Late Miocene.
- Mid- to Late Miocene AHe cooling ages from the Valsugana thrust, the Val Trompia thrust, the Tonale fault and the Giudicarie belt in the Southern Alps indicate at least 2 km of exhumation since then. Along thrust systems (i.e., Valsugana and Val Trompia thrusts) uplift and erosion were larger as demonstrated by Miocene AFT ages.
- However, even along the thrusts exhumation can be highly differential, as shown for the western sector of the Valsugana thrust.
- Exhumation and deformation related to Adria indentation was initially confined within the orogenic core of the Eastern Alps, the Tauern Window, but became more widespread during Mid- to Late Miocene times.
- This shift from focused exhumation outside the indenter to exhumation within, and deformation of the indenter, is ascribed to a major shift in the coupling state, from a decoupled to a coupled system.

## Electronic supplementary material

Below is the link to the electronic supplementary material.

Supplementary material 1 (DOCX 28 kb)
