# Supplementary material for: Postcollisional cooling history of the Eastern and Southern Alps and its linkage to Adria indentation
Source: Int J Earth Sci. 2016 Jul 13;106(5):1557–80. doi: 10.1007/s00531-016-1367-3 (PMC6979704; doi:10.1007/s00531-016-1367-3)
Supplement: Supplementary file 1 — Supplementary material 1 (DOCX 28 kb) [file 531_2016_1367_MOESM1_ESM.docx]

**Supplementary file**

Postcollisional cooling history of the Eastern and Southern Alps and its linkage to Adria indentation

Bianca Heberer, Rebecca Lee Reverman, Maria Giuditta Fellin, Franz Neubauer, István Dunkl, Massimiliano Zattin, Diane Seward, Johann Genser and Peter Brack

**Corresponding author:**

Bianca Heberer

Dept. Geography and Geology

University of Salzburg

Hellbrunner Str. 34

5020 Salzburg, Austria

E-Mail: bianca.heberer@sbg.ac.at

Phone: ++43-662-8044-5461

Fax: ++43-662-8044-621

**Methodology section for apatite fission track analyses and (U-Th-Sm)/He dating on apatites**

For mineral separation, bulk rock samples of approximately 5 kg were crushed, milled and sieved. The grain fraction between 250µm and 63µm was put through density separation and magnetic separation to obtain a pure apatite separate. For **fission-track analysis** a small aliquot of apatite (n =200) was put into a teflon form and covered in epoxy. At least 2 mounts were made for each sample. The mounts were put into a vacuum chamber to remove all air bubbles, and then put into an oven at 50 °C for 2-3 hours to set the epoxy. After 24 hours curing of the epoxy was complete and mounts of grains were polished and etched in 5.5N HNO_3_ at 21 °C for 20 seconds to reveal spontaneous tracks. Low U muscovite sheets (the external detector) were placed and taped tightly against the polished side of the grain mounts. Mounts plus at least one standard were loaded into plastic containers. Dosimeters (CN- 5) were placed at either end of each sample stack to measure neutron fluence. Samples were irradiated with thermal neutrons in the reactor at the Radiation Center of Oregon State University with a nominal fluence of 1x10^16^n cm^-2^. After irradiation, induced fission tracks in the external detectors were exposed through etching in 40% HF at 21 °C for 45 minutes. Apatite FT ages were calculated using the external-detector and the zeta-calibration methods (Hurford and Green 1983) with IUGS standards and a value of 0.5 for the 4π/2π geometry correction factor. Counting took place at a magnification of 1250x. The analyses were subjected to the χ^2^-test (Galbraith 1981) to detect whether the data sets contained any extra-Poissonian error. A χ^2^-probability of less than 5% denotes a significant spread of single grain dates.

Apatite crystals for **AHe analysis** were hand-picked following the selection criteria of Farley (2002). Euhedral crystals were inspected for inclusions under 250x magnification and cross-polarized light. Only inclusion-free grains were selected. To calculate the alpha ejection correction factor (Farley et al. 1996) microphotographs were taken for determining external dimensions (width, total length, and length of prismatic section). Analyses of the Austroalpine Karawanken samples were carried out in the Thermochronology Laboratory at Geoscience Center, University of Göttingen, Germany. Measurements of the Southalpine samples were done at the Noble Gas Lab at ETH-Zürich. After proper documentation, each crystal was loaded in ca. 1x1 mm platinum capsules and degassed in high vacuum by heating with an infrared laser. The ^4^He content of the purified gas extracted from the crystals was measured on a Hiden® triple-filter quadrupole mass spectrometer in Göttingen and on a sector gas single collector mass spectrometer (Albatross) in Zurich.

For the detection of the alpha-emitting elements (U, Th and Sm) the degassed crystals were spiked with calibrated ^230^Th and ^233^U solutions (Göttingen) / ^233^U, ^236^U, ^230^Th and ^149^Sm (Zürich). After dissolution, the actinide concentrations were measured on ICP quadrupole mass spectrometers.

**References**

Farley KA (2002) (U-Th) ⁄He dating: Techniques, calibrations, and applications. Mineral Soc Am Rev Mineral Geochem 47:819-844. doi: 10.2138/rmg.2002.47.18

Farley KA, Wolf RA, Silver LT (1996) The effects of long alpha-stopping distances on (U-Th)/He ages. Geochim Cosmochim Acta 60:4223-4229. doi: 10.1016/S0016-7037(96)00193-7

Galbraith RF (1981) On statistical models for fission track counts. J Int Assoc Math Geol 13:471-478. Doi: 10.1007/BF01034498

Hurford AJ, Green PF (1983) The zeta age calibration of fission-track dating. Chem Geol 41:285-317. [doi:10.1016/S0009-2541(83)80026-6](http://dx.doi.org/10.1016/S0009-2541%2883%2980026-6)
